# Curative effect of external fixation combined with kirschner wire versus hollow screw in the treatment of first metacarpal bone base fracture

**DOI:** 10.1186/s12891-023-06938-1

**Published:** 2023-10-23

**Authors:** Jian Liu, Zhengfeng Lu, Gang Zhao, Yuzhou Liu, Haoyu Yang, Mingyu Xue

**Affiliations:** Department of Orthopedics, Wuxi 9th People’s Hospital Affiliated to Soochow University, No.999 Liangxi Road, Binhu District, Wuxi, 214000 Jiangsu China

**Keywords:** External fixation, Kirschner wire fixation, Hollow screw fixation, Curative effect

## Abstract

**Purpose:**

This study was conducted aimed at comparing the curative effect of external fixation combined with Kirschner wire fixation versus hollow screw fixation in the treatment of first metacarpal bone base fracture.

**Methods:**

The current retrospective study included a total of 80 patients diagnosed with first metacarpal bone base fracture who were admitted in Wuxi 9th People’s Hospital Affiliated to Soochow University between October 2017 and October 2020. The patients enrolled were equally divided into the combined group (40 cases, receiving external fixation combined with Kirschner wire fixation), and the control group (40 cases, receiving hollow screw fixation). Perioperative indices were collected and compared between the two groups. Pain scores before operation and three months, six months, and one year after operation were compared. Additionally, we compared the finger function in the last follow-up visit ( the follow-up period was 1 year) and rate of complications.

**Results:**

Operation time, amount of bleeding, length of incision, length of hospital stay, and fracture healing time did not differ between the two groups (all *P* > 0.05). Pain score was comparable between the two groups before operation (*P* = 0.704). Despite lower results showing at 3, 6, and 12 months after operation in both groups, the pain score did not significantly differ in any time point between the two groups (all *P* > 0.05). Additionally, no significant differences were observed in finger function and rate of complications at the last follow-up between the two groups (both *P* > 0.05).

**Conclusion:**

External fixation combined with Kirschner wire fixation and hollow screw fixation exhibited similar curative effect in treating first metacarpal bone base fracture, indicating both surgery methods may be considered as the preferred approach.

## Introduction

Metacarpal fracture are a common injury, accounting for 36-42% of hand trauma cases in men aged 18–34 [1]. The first metacarpal bone is affected in around 25% of all metacarpal fractures, with 80% of those fractures occurring at the base [2, 3]. If a metacarpal fracture is not properly managed, malunion may occur, leading to pain, stiffness and loss of grip strength in the finger joint, which can have a serious impact on the quality of life of patients [4]. As a result, it is crucial to use appropriate treatment methods when treating fracture at the base of the first metacarpal bone.

Non-surgical methods were previously applied to treat fracture at the base of the first metacarpal bone. However, the presence of muscle tissues such as abductor pollicis brevis, flexor pollicis brevis, abductor pollicis longus and extensor pollicis longus around the base of the first metacarpal can result in fracture end instability, fracture redisplacement, and bone nonunion without internal fixation. Moreover, the thumb plays a vital role in hand function, and inadequate treatment can significantly impair hand function. Therefore, surgery has become the preferred for this type of fracture in recent years [5]. Surgical options include small splint fixation, conventional manual reduction, plaster fixation and plate or Kirschner wire internal fixation. However, the implantation of plate can lead to increased tension in skin sutures and is prone to exposed in the event of infection and necrosis after operation [6]. Meanwhile, the simple use of Kirschner wire is often insufficient to achieve stable fixation, and the displacement of articular surface fracture block and articular surface malunion are common post-operative complications. As such, alternative surgical methods are urgently needed to effectively treat fracture at the base of first metacarpal bone .

The use of hollow screw fixation can partially overcome the drawbacks of Kirschner wire fixation, such as easy loosening and infection, and has been proven effective in treating metacarpal fracture [7]. However, there is limited literature on the use of hollow screw fixation in treating first metacarpal bone base fracture. External fixation combined with Kirschner wire fixation can effectively improve stability without causing extensive damage to the ligament or compromising the blood supply at the fracture site, thereby preserving the integrity of the surrounding soft tissue [8]. This approach may provide a new option for treating first metacarpal bone base fracture [9]. Therefore, this study was conducted to compare the curative effect of external fixation combined with Kirschner wire fixation versus hollow screw fixation in the treatment of first metacarpal bone base fracture.

## Methods

### Study design

The present study was designed as a retrospective clinical study. The study was conducted in accordance with the requirements of the Declaration of Helsinki and was approved by the Ethics Committee of Wuxi 9th People’s Hospital Affiliated to Soochow University. Informed consent was obtained from the subjects.

### Study subjects

Patients with first metacarpal bone base fracture who were admitted in Wuxi 9th People’s Hospital Affiliated to Soochow University were enrolled in this study. After informing the patients about the specific surgical procedures and risks, the patients were allowed to choose the surgical method themselves. Ultimately, a total of 80 cases were included, with 40 patients treated with external fixation combined with Kirschner wire fixation in the combined group. The combined group included 12 cases of type I (Bennett fracture), 18 cases of type II (Rolando fracture), and 10 cases of type III (extraarticular fracture). The control group consisted of 40 patients who underwent hollow screw fixation, including 14 cases of type I (Bennett fracture), 17 cases of type II (Rolando fracture), and 9 cases of type III (extraarticular fracture).

In order to be included in the current study, the following inclusion criteria should be met: (1) unilateral fracture; (2) patients who were diagnosed with first metacarpal bone base fracture using X-ray examination; (3) non-pathological fracture; (4) patients without surgical contraindications; (5) informed consent were obtained from patients or guardians. Exclusion criteria were as follows: (1) pathological fracture or old fracture (more than 3 weeks); (2) no CT study of the trapezius, scaphoid or first metacarpus; (3) thickness of scanning layer ≥ 7 mm; (4) the first carpal metacarpal joint deviated significantly from the rest position; (5) patients with previous trauma and operation history of the ipsilateral upper limb or same finger; (6) patients who were accompanied by rupture of blood vessels, nerves and tendons; (7) open fracture; (8) patients who were lost to follow-up.

### Surgery methods

The control group patients underwent hollow screw fixation. After satisfactory brachial plexus block anesthesia, the base of the metacarpal bone was pressed for fracture reduction. C-arm machine fluoroscopy was used to confirm satisfactory reduction. The metacarpophalangeal joint was flexed to 90° and pulled to maintain the fracture position at the same time. After reduction, a Kirschner wire was then inserted into the vertical fracture line under fluoroscopy, followed by an obliquely inserted 0.8 mm guide needle into the metacarpal head. The skin around the guide needle was cut by approximately 0.5 cm, and the skin, subcutaneous tissue, and deep fascia were cautiously separated to avoid the extensor tendon. A 1.7 mm hollow screw (TriMed, CA, USA) was then screwed in. Kirschner wire and guide needle were removed. Simple fractures were treated with closed reduction and hollow screw fixation, while open reduction of non-pathological fractures was performed using the Wagner approach [10].

The combined group patients underwent external fixation combined with Kirschner wire fixation. Firstly, two 2.0 mm diameter self-tapping thread needles were vertically drilled in the middle distal of the first metacarpal bone after satisfactory brachial plexus block anesthesia, followed by one or two 2.0 mm diameter self-tapping thread needles that were drilled at 45° degree angle on the plane of the trapezium and dorsum of the hand. Care was taken to avoid the abductor pollicis longus tendon and extensor pollicis brevis tendon. The bone holding clamp and bolt were installed, and the thumb outside the booth was pulled while the base of the metacarpal bone was pressed for fracture reduction. After the fracture reduction was confirmed by C-arm fluoroscopy, the position of thumb abductor and thumb opposition was maintained, and the bone holding clamp was locked. If the reduction of the fracture block was difficult during the operation, a small arc incision was made at the base of the first metacarpal on the radial dorsal side, and extensor pollicis brevis tendon and extensor pollicis longus tendon were retracted. Direct vision was used to expose and cut the first carpal metacarpal capsule, and the fracture was reduced by prying. One or two Kirschner wires with a diameter of 1.0-1.2 mm were fixed, and the bone holding clamp was locked after confirming the fracture reduction via fluoroscopy. Finally, plaster external fixation was performed.

Patients in both groups received rehabilitation training. Active movements of the metacarpophalangeal and interphalangeal joints were initiated on the second day after the surgery. After 72 h post-operation and pain relief, active functional exercises such as flexion and extension of the first carpometacarpal joint, abduction of the first carpometacarpal joint, and opposition to the palm, finger, and fist were gradually performed. X-ray images were reviewed every 1–2 months to monitor the progress of the healing process.

### Observational indices

Perioperative indices, including operation time, amount of bleeding, length of incision, length of stay, fracture healing time were collected and compared. Pain scores before operation and 3, 6, and 12 months after the operation were also evaluated based on a *visual analogue scale in pain evaluation* [11]. During the one-year follow-up period, we compared finger function and the rate of complications at the last follow-up. Finger function was evaluated using the total angle of motion (TAM) based on trial criteria for upper limb function assessment of the Hand Surgery Society of Chinese Medical Association [12]. We defined four levels of finger function recovery: normal finger movement (recovery), TAM > 75% of healthy side (excellent effect), TAM of 50-75% of healthy side (effectiveness), and TAM < 50% of healthy side (ineffectiveness).

### Statistical analysis

All statistical analyses were performed with the use of SPSS V.21 software. Countable data of normal distribution was presented as mean ± standard deviation, while data failing to conform the normal distribution was presented as median and quartiles. Comparison of countable data between the two groups were performed using t test or Mann-Whitney U test. Categorical data was presented as number or percent. Additionally, we compared the categorical data using Chi-square test. A *P* < 0.05 indicated significant difference.

## Results

### Baseline characteristics of participants

**Fig. 1:**
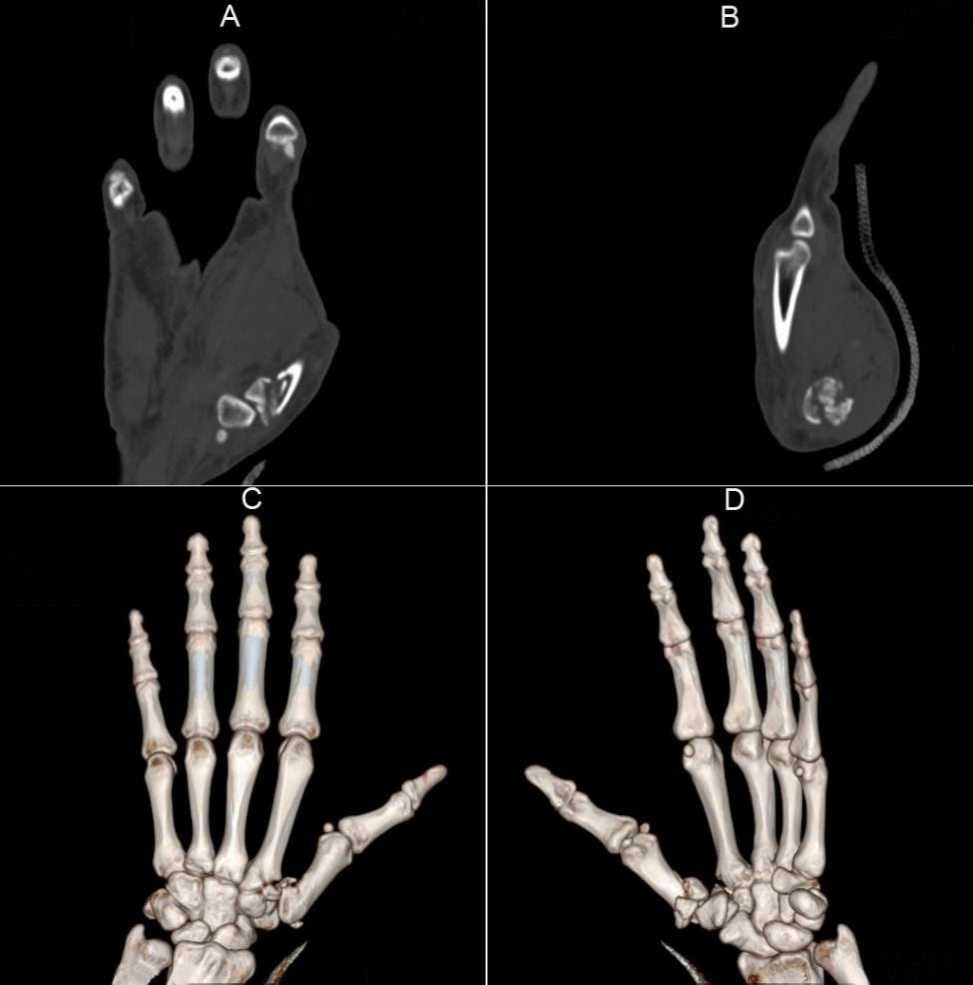
Representative computed tomography images of patients with communited first metacarpal bone base fracture. (**A** and **B**) The fracture of the first metacarpal bone base led to comminuted articular surface, partial articular surface collapse, and dorsal dislocation of the first carpometacarpal joint. (**C** and **D**) The fracture of the first metacarpal bone base led to that the joint was displaced to the palmar side and was crushed into three pieces

In the combined group, there were a total of 24 males and 16 females with ages ranging from 25 to 71 years old, and a mean age of 49.98 ± 11.28 years old. Meanwhile, the control group consisted of 22 males and 18 females with ages ranging from 24 to 70 years old, and a mean age of 49.78 ± 11.78 years old. Both groups had similar body mass index (BMI), with the combined group ranging from 18 to 26 kg/m^2^ and the control group ranging from 18 to 25 kg/m^2^. The mean BMI in the combined group was 23.01 ± 2.23 kg/m^2^, while it was 22.93 ± 2.19 kg/m^2^ in the control group. As for the types of injuries sustained by patients in the combined group, 9 cases suffered fist injury, 13 cases suffered machine hurt, and 18 cases suffered fall damage. In comparison, the control group had slightly more cases of each type of injury, with 10 cases of fist injury, 14 cases of machine hurt, and 16 cases of fall damage. Some patients suffered from complex fractures, including two cases with comminuted fractures of the first metacarpal bone base. One of these cases resulted in comminuted articular surface, partial articular surface collapse, and dorsal dislocation of the first carpometacarpal joint (Fig. 1A and B). The other case led to the displacement of the joint to the palmar side and the bone being crushed into three pieces (Fig. 1C and D). The time from injury to operation in both the combined and control groups ranged from two to seven days, with mean values of 3.28 ± 0.78 days and 3.22 ± 0.75 days, respectively. There were no significant differences between the two group in any of these indices.

### Perioperative indices between the two groups

The combined group and control group had mean operation time of 21.21 ± 3.87 min and 20.82 ± 3.78 min, respectively, while the mean amount of bleeding was 26.87 ± 3.76 mL and 25.76 ± 3.87 mL, respectively. The mean length of incision in the two groups were 3.02 ± 0.87 cm and 2.69 ± 0.68 cm, respectively, and the mean length of stay was 4.28 ± 1.02 days and 4.13 ± 1.05 days, respectively. The mean fracture healing time in the two groups were 6.91 ± 2.31 and 6.73 ± 2.13 weeks, respectively. There were no significant differences between the two groups in any of these perioperative indices (all *P* > 0.05, Table 1).

**Table 1:** Comparison of perioperative indicators between the two groups

Indices	Combined group (n = 40)	Control group (n = 40)	t value	*P* value
Operation time (min)	21.21 ± 3.87	20.82 ± 3.78	0.456	0.651
Amount of bleeding (mL)	26.87 ± 3.76	25.76 ± 3.87	1.301	0.197
Length of incision (cm)	3.02 ± 0.87	2.69 ± 0.68	1.891	0.062
Length of stay (days)	4.28 ± 1.02	4.13 ± 1.05	0.648	0.519
Fracture healing time (weeks)	6.91 ± 2.31	6.73 ± 2.13	0.362	0.718

Data was presented as mean ± standard deviation. *P* < 0.05 was considered of significant difference

### Relief of pain after operation between the two groups

There were no significant differences of pain score before operation between the two groups (*P* = 0.704). Pain scores were lower in both groups after operation, with scores of 2.15 ± 0.41 and 2.07 ± 0.35 at 3 months, 1.37 ± 0.39 and 1.27 ± 0.32 at 6 months, and 1.02 ± 0.28 and 0.93 ± 0.24 at 12 months for the combined and control groups, respectively. No significant differences were observed in pain scores between the two groups at any time point (all *P* > 0.05, Table 2).

**Table 2:** Comparison of pain score between the two groups

Time points	Combined group (n = 40)	Control group (n = 40)	t value	*P* value
Pre-operation	7.92 ± 1.23	7.81 ± 1.35	0.381	0.704
Three months after operation	2.15 ± 0.41	2.07 ± 0.35	0.939	0.351
Six months after operation	1.37 ± 0.39	1.27 ± 0.32	1.254	0.214
One year after operation	1.02 ± 0.28	0.93 ± 0.24	0.686	0.495

Data was presented as mean ± standard deviation. *P* < 0.05 was considered of significant difference

### Recovery of finger function between the two groups

**Fig. 2:**
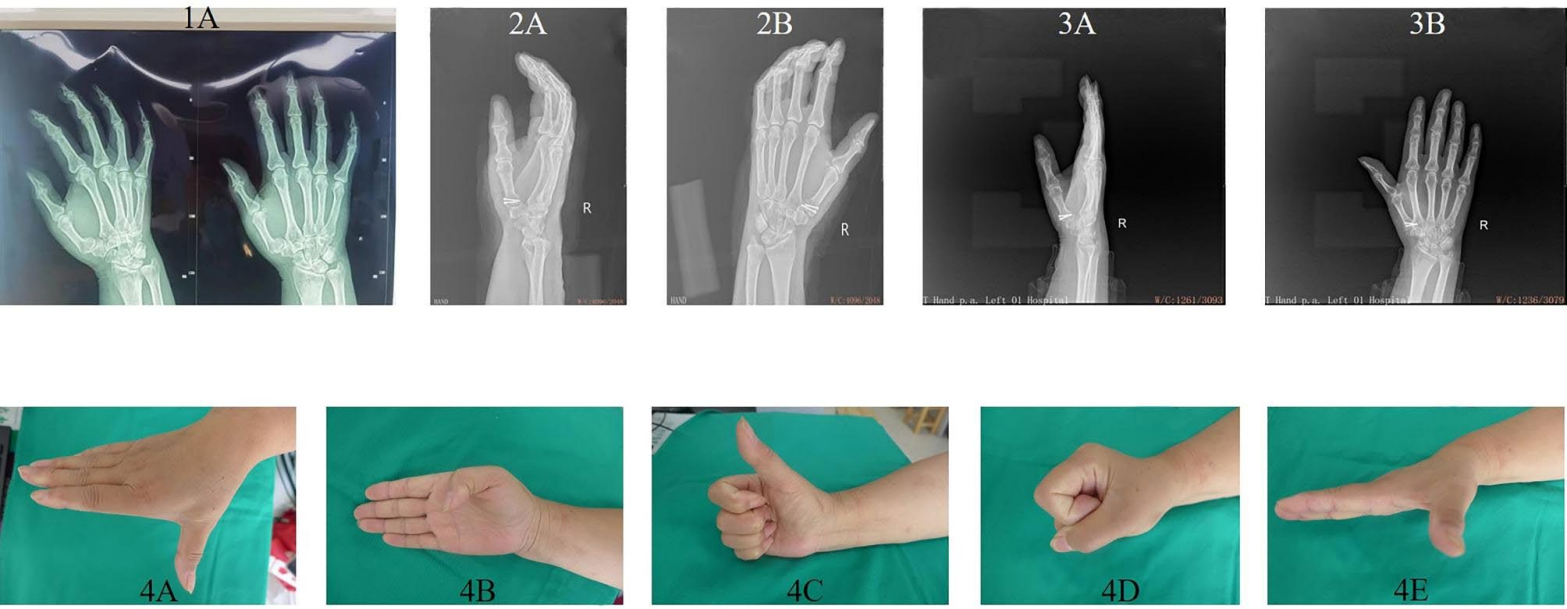
Representative clinical treatment course of a patients treated with hollow screw fixation. The patient suffered first metacarpal bone base fracture because of fall damage. (1 **A**) X-ray examination before operation showed first metacarpal bone base fracture. (2 **A** and 2**B**) X-ray examination after hollow screw fixation showed that the first carpometacarpal joint surface was restored, and the first carpometacarpal joint was in position. The fixation was fixed for 4 weeks after operation, and functional exercise was started after 4 weeks. (3 **A** and 3**B**) X-ray examination two months after operation showed that the fracture healed. (4 **A**-**E**) The function recovered well at 8 months after operation, with no joint movement pain and limitation

**Fig. 3:**
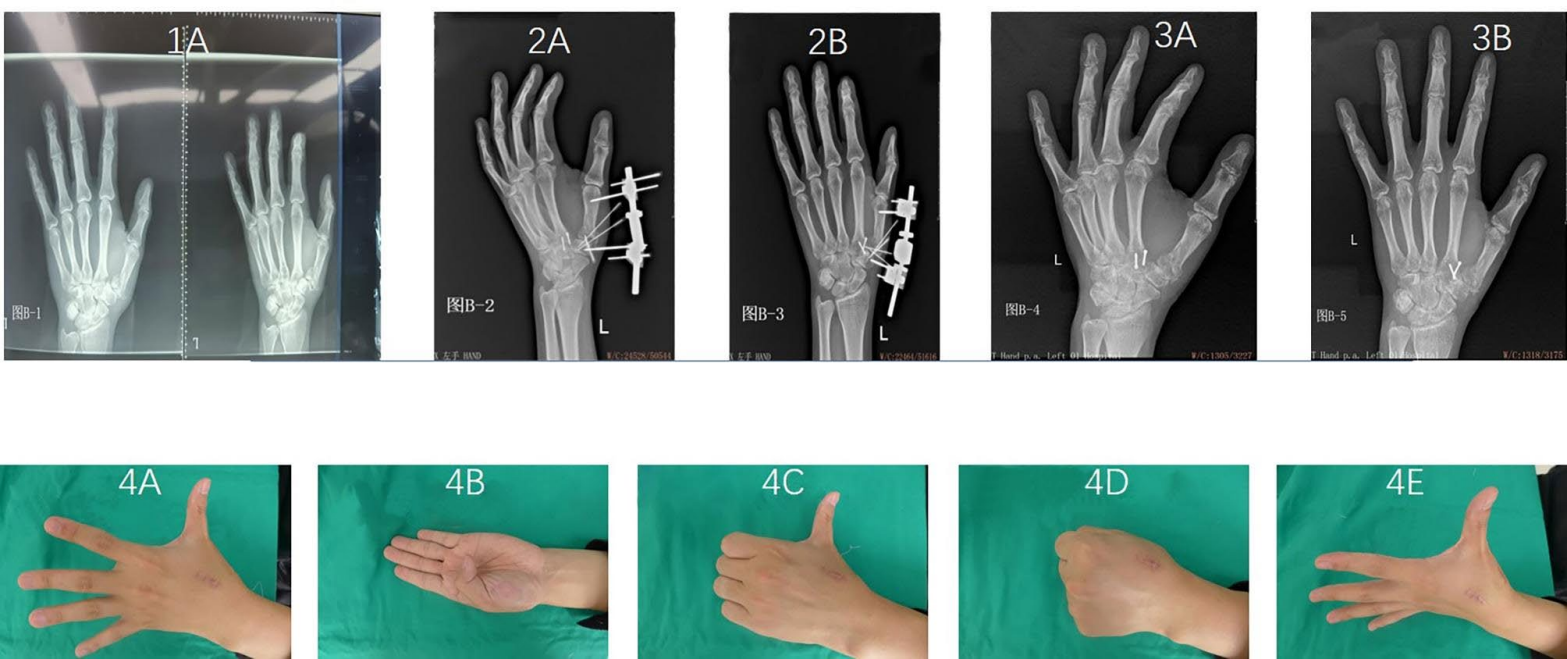
Representative clinical treatment course of a patients treated with external fixation combined with Kirschner wire fixation. The patient suffered comminuted base fracture of first metacarpal bone because of pressed mark by a heavy object. (1 **A**) X-ray examination before operation showed comminuted base fracture of first metacarpal bone. (2 **A** and 2**B**) X-ray examination after operation showed that the first carpometacarpal joint surface was restored, and the first carpometacarpal joint was in position. The fixation was fixed for 4 weeks after operation, and functional exercise was started after 4 weeks. (3 **A** and 3**B**) X-ray examination six weeks after operation showed that the external fixation and Kirschner wire were removed. (4 **A**-**E**) The function recovered well after operation, with no joint movement pain and limitation

At the last follow-up, 20 patients in the combined group and 17 patients in the control group fully recovered finger function. The surgery had excellent effects on 13 patients in the combined group and 12 patients in the control group, and was effective in 6 patients in the combined group and 11 patients in the control group. However, the surgery was ineffective in one patient in the combined group. Figures 2 and 3 depict representative case treated with external fixation combined with Kirschner fixation and hollow screw fixation. The combined and control groups had effective rates of 97.50% and 100.00%, respectively, with no significant difference in recovery of finger function between the two groups (χ^2^ = 1.013, *P* = 0.314) (Table 3).

**Table 3:** Comparison of post-operative recovery of finger function between the two groups [n (%)]

Indices	Combined group (n = 40)	Control group (n = 40)	χ^2^ value	*P* value
Effective rate	39 (97.50)	40 (100.00)	1.013	0.314
Recovery	20 (50.00)	17 (42.50)		
Excellent effect	13 (32.50)	12 (30.00)		
Effectiveness	6 (15.00)	11 (27.50)		
Ineffectiveness	1 (2.50)	0 (0.00)		

*P* < 0.05 was considered of significant difference

### Rate of complications between the two groups

**Fig. 4:**
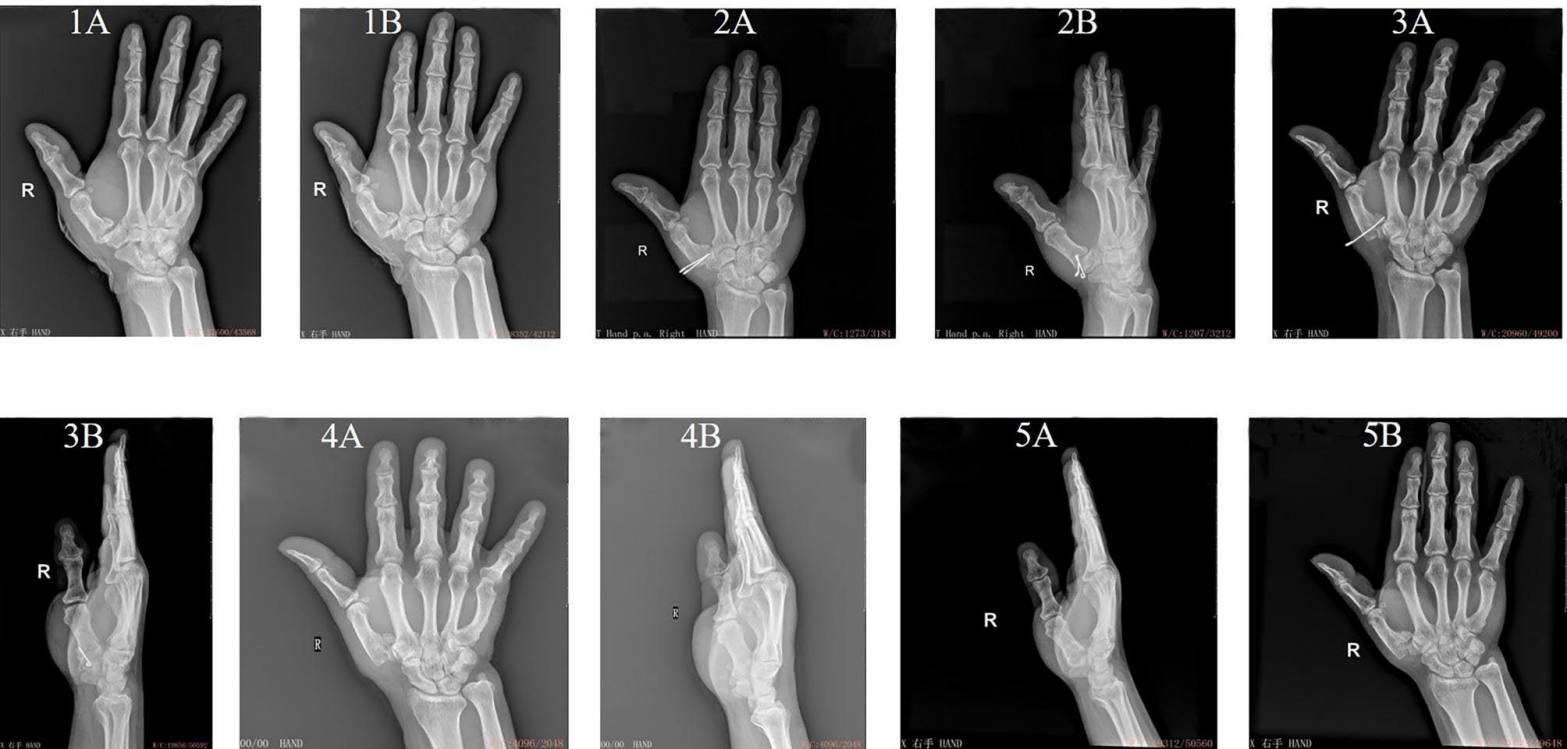
Representative clinical treatment course of a patients treated with external fixation combined with Kirschner wire fixation and suffered complications after operation. (1 **A** and 1**B**) X-ray examination before operation showed comminuted base fracture of first metacarpal bone. (2 **A** and 2**B**) X-ray examination one month after operation showed that the first carpometacarpal joint surface was restored, and the first carpometacarpal joint was in position. (3 **A** and 3**B**) X-ray examination showed that the loosening of external fixation without timely adjustment resulted in fracture displacement and internal fixation failure. (4 **A**-**B**) X-ray examination showed that the Kirschner wires were removed. (5 **A**-**B**) X-ray examination showed that the first carpometacarpal joint surface was restored, and the first carpometacarpal joint was in position finally

The follow-up period in both groups ranged from one year to 14 months, with a mean value of 12.87 ± 0.25 months. One patient in the combined group suffered pin-track infection and fracture displacement, as shown in Fig. 4. No patients in the control group suffered complications. No significant difference was found in rate of complications between the two groups (χ^2^ = 2.051, *P* = 0.152)(Table 4).

**Table 4:** Comparison of post-operative complications between the two groups [n (%)]

Complications	Combined group (n = 40)	Control group (n = 40)	χ^2^ value	*P* value
Total incidence	2 (5.00)	0 (0.00)	2.051	0.152
Pin-track infection	1 (2.50)	0 (0.00)		
Incision infection	0 (0.00)	0 (0.00)		
Fixator breakage or loosening	0 (0.00)	0 (0.00)		
Breakage or loosening of external fixation screw	0 (0.00)	0 (0.00)		
Fracture displacement	1 (2.50)	0 (0.00)		

*P* < 0.05 was considered of significant difference

## Discussion

This study demonstrated that both surgical methods are viable treatment options for first metacarpal bone base fracture, as they showed comparable perioperative indices, relief of pain after operation, recovery of finger function at the last follow-up, and rate of complications between the combined group and control group.

Fractures of the first metacarpal bone base are complex due to the large range of motion of the first carpometacarpal joint and its susceptibility to axial and tangential external forces. This often results in various types of fractures and dislocations caused by joint stability structure damage [13]. Additionally, due to the traction of different ligaments and muscles attached to the fracture fragment of the first metacarpal bone base, various displacements and deformities may occur, making it challenging to maintain reduction and reliable fixation [13]. The treatment of hand fractures requires anatomical reduction as much as possible, strong internal fixation, and early functional exercise to reduce the interference from fixation devices on soft tissues and carpometacarpal joints and restore the function of metacarpophalangeal joints [14]. A range of internal fixation materials and methods are available in treating metacarpophalangeal fractures, including absorbable screw rods, hollow screws, steel plate screws, Kirschner wires, steel wires, etc. Recent studies have suggested that Kirschner wires and hallows screws are commonly used [15]. In the treatment of fractures, hollow screw fixation has the characteristics of being cost-effective, providing firm fixation, little influence on joints, enabling early functional exercise, good postoperative joint activity, and rapid recovery of function [16]. However, some patients may be complicated with carpometacarpal joint osteoarthritis, the occurrence of carpometacarpal joint osteoarthritis is associated with joint degeneration, strain and trauma, and its pathological mechanism may be degenerative changes in the articular cartilage surface, the formation of osteophytes, resulting in abrasive pain and significantly reduced pinch power and other related symptoms. Therefore, given that most metacarpophalangeal fractures are unstable fractures, using only Kirschner wire internal fixation can lead to complications such as rotation, which may cause detachment of the broken end of the reduction fracture [17]. External fixation combined with Kirschner wire fixation may achieve good surgical results. Peng et al. [18] found that the combination of external fixation and Kirschner wire fixation can effectively treat closed fracture or dislocation of the fifth metacarpal base, resulting in remarkable therapeutic effects. In their study, the approach has several advantages, such as minimal trauma, simple operation, and reliable fixation. Li et al. [19] concluded that mini external fixation combined with Kirschner wire fixation offered efficient treatment for open comminuted metacarpophalangeal joint fractures, which is easy to operate, provides good stability, allows for later adjustment and early functional exercise, and causes minimal damage to soft tissue and periosteum, with low infection rate. In this study, we used hollow screw as the control and observed no significant differences in several factors, including operation time, amount of bleeding, length of incision, length of hospital stay, and fracture healing time between the combined group and the control group. These findings suggested that external fixation combined with Kirschner wire fixation caused minimal injury and preserved the blood supply of the fracture end, thus achieving good surgical outcomes.

The clinical symptom that is most evident in patients with a fracture is severe pain at the site of fracture, which is often accompanied by significant discomfort following surgery. Therefore, a key focus in clinical practice involves carefully observing the degree of pain experienced by patients with first metacarpal base fractures. The results of this study showed that at 3, 6 and 12 months after operation, the pain scores of both groups were lower than those before surgery. Furthermore, the pain scores of the two groups were comparable, providing evidence to support the idea that a combination of external fixation and Kirschner wire fixation can effectively reduce the postoperative pain in these patients.

The study conducted by Zhong et al. [20] indicated that the combination of mini external fixator and Kirschner wire internal fixation exhibited a good effect in treating metacarpophalangeal joint fractures, resulting in better recovery of the function of joint after operation. Liu [21] reported significant clinical value in using this combination for treating closed proximal phalangeal fractures, achieving good reduction effect and significant recovery of function with shorter rehabilitation time. The present study demonstrated that both groups achieved comparable function of the affected fingers after surgery, indicating that external fixation combined with Kirschner wire fixation can effectively restore the function of the affected fingers.

Tang et al. [22] found that there were no postoperative complications in the treatment of the first metacarpal base fracture with external fixation combined with Kirschner wire fixation. Consistently, this study further supported the safety of this approach, as the rate of complications was comparable in both groups. However, it should be noted that one patient in the combined group experienced a loosening of the external fixation, which was not treated timely and caused a shift of the fracture. Additionally, pin-track infection occurred due to wetness at the pin-track site.

Based on the results of this study and the findings from previous literature reports, [23, 24], we can conclude that external fixation combined with Kirschner wire fixation offers several advantages: (1) It is a minimally invasive operation that promotes fracture healing without damaging the blood supply of the fracture end by avoiding periosteal stripping; (2) in cases where fracture reduction is challenging or unsatisfactory, Kirschner wire pocking reduction can be performed through a small incision; (3) it can open the carpometacarpal joint, maintain the normal joint space, effectively prevent the contracture of joint capsule and collateral ligament, and thus improve joint function recovery after operation while reducing the risk of traumatic arthritis; (4) the surgical incision is small, making it suitable for children and young women; (5) it does not require the removal of internal fixation and, therefore, avoids any secondary surgical trauma. However, it is essential to note that the strength of external fixation combined with Kirschner wire fixation is inferior to that of the hollow screw fixation. [25] The main advantages of using hollow screw fixation are as follows [26, 27]: (1) hollow screw is drilled through Kirschner wire guide, which causes less damage to the peripheral blood supply and the soft tissue surrounding the fracture. Also, this method reduces the likelihood of causing trochlear necrosis during the surgery, which is beneficial for postoperative fracture healing; (2) due to the threaded structure at the top of the cannulated screw, the broken ends can be brought into close contact by compression, leading to better anatomical reduction of the articular surface, firm fixation, and earlier fracture healing than Kirschner wire fixation; (3) the internal fixation has little effect on the joint, enabling postoperative joint movement, and relatively reliable fixation, which is conducive to early postoperative rehabilitation; (4) there is minimal impact of the hollow screw on the tendon, which helps to avoid tendon wear and rupture. However, some disadvantages are associated with this method, such as the inability to use a hollow screw if the fracture is too small. In any case, both methods are considered preferred treatment for first metacarpal bone base fracture, and surgeons can determine the most appropriate treatment option based on their proficiency in surgery techniques. In addition, in our experience, if the patient’s skin and soft tissue are good and the fracture mass is large, the external fixation combined with hollow screw treatment is preferred.

This study had some limitations, including the limited sample size which prevented the observation of possible complications such as soft tissue irritation and flap necrosis. And the design of follow-up needs to be more scientifically rigorous and detailed. Moreover, the results presented in this study should be further confirmed by additional biomechanical experimental data. In addition, Kapandji score and MHQ (Michigan Hand Outcomes Questionnaire, self-assessment questionnaire of patients with hand and wrist diseases) were not used to evaluate patients in this study. Further research will focus on the above indicators.

## Conclusion

Both external fixation combined with Kirschner wire fixation and hollow screw fixation are considered preferred surgery methods for first metacarpal bone base fracture.

## Data Availability

The datasets generated and analyzed during the current study are available from the corresponding author on reasonable request.
